# Beyond the Healthdot: Insights from Three Years of Same-day Discharge and Remote Monitoring in Bariatric Surgery

**DOI:** 10.1007/s11695-026-08557-w

**Published:** 2026-03-04

**Authors:** Kayleigh A.M. van Dam, Jeanine Leenen, Pieter P.H.L. Broos, Evelien de Witte, Esther P.W.A. Talboom-Kamp, Evert-Jan G. Boerma

**Affiliations:** 1 Bariatric Surgery, Zuyderland Medisch Centrum, Heerlen, Netherlands; 2 NUTRIM, Maastricht University Medical Centre, Maastricht, Netherlands; 3 Department of Business Intelligence, Zuyderland Medisch Centrum, Heerlen, Netherlands; 4 NOK Zuid, Nederlandse Obesitas Kliniek, Heerlen, Netherlands; 5 Faculty of management science, Open University in the Netherlands, Heerlen, Netherlands

## Abstract

• Same-day discharge is safe and feasible in primary Metabolic Bariatric Surgery.

• Analysis of 679 patients with Healthdot monitoring after same-day discharge revealed no consistent predictive trends.

• The complete perioperative care pathway, including eNurse contact, may be key to patient satisfaction and safety.

## Introduction

Same-day discharge following Metabolic Bariatric Surgery (MBS) has emerged as a safe and feasible practice, as demonstrated in our previous study [1]. As a follow-up, we retrospectively evaluated a cohort of 697 patients who underwent primary MBS between March 2022 and March 2025 in a large Dutch teaching hospital. All patients were continuously monitored postoperatively using the Healthdot for seven days [ref website Healthdot]. This follow-up study aimed to explore whether remote monitoring could reliably predict postoperative complications and whether current threshold values should be adjusted.

## Methods

Eligibility for same-day discharge and Healthdot monitoring was determined according to predefined criteria, described in our previous study [1]. These criteria excluded patients with significant endocrinologic, cardiovascular or pulmonary disease, those using anticoagulants or rhythm-control medication, and those with an implantable cardioverter defibrillator (ICD) or pacemaker. Additionally, patients were required to reside within a maximum travel time of 30 min from the hospital.

**Fig. 1:**
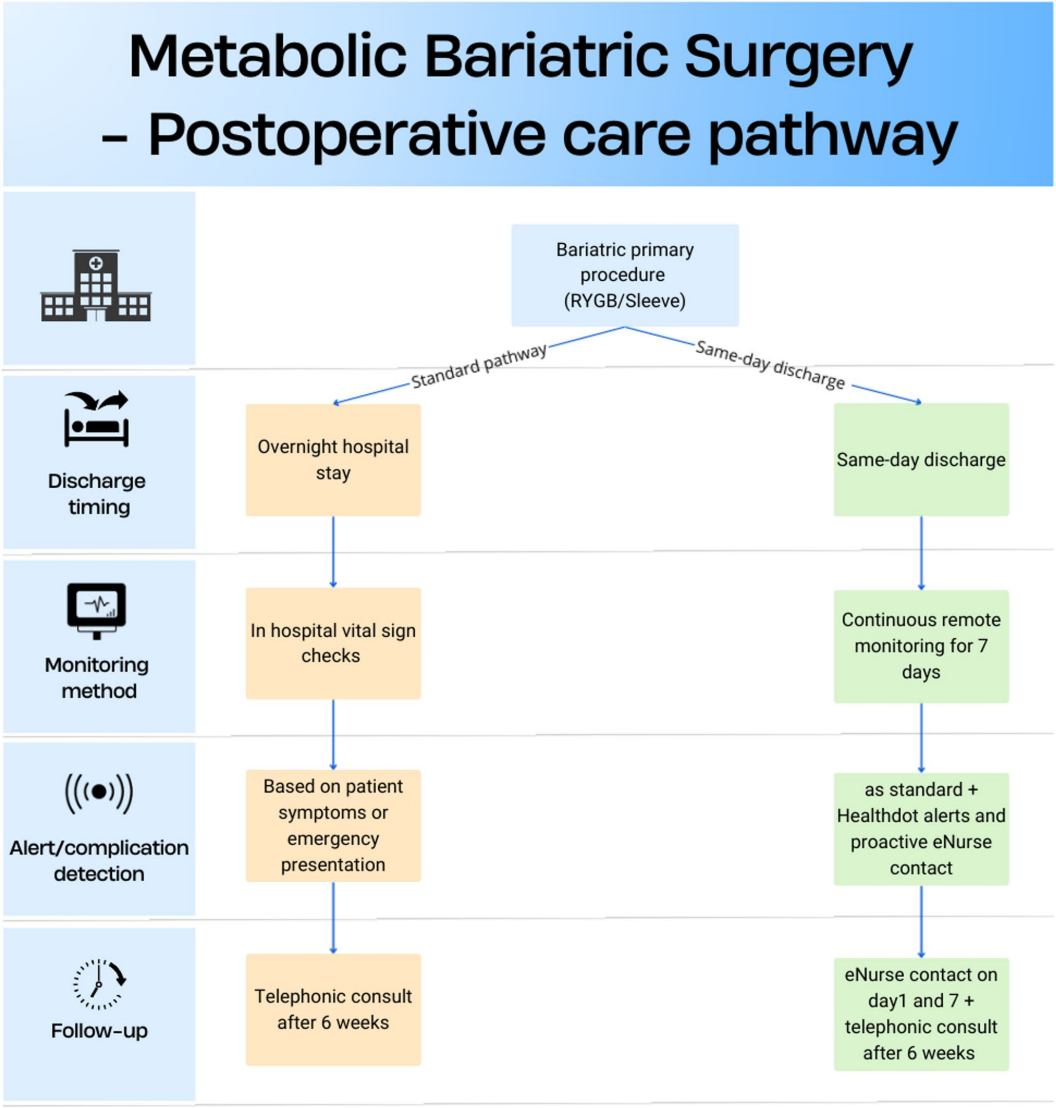
Comparison of standard vs. same-day discharge care pathways following metabolic bariatric surgery

Beside the same-day discharge, the bariatric surgery care pathway includes structured follow-up with telephone calls by an eNurse on postoperatively day 1 and 7. These calls provide patients with an opportunity to report symptoms, receive reassurance and discuss medication use. In contrast, the standard pathway involves an overnight hospital stay and a single telephonic consult at six weeks, without early structured follow-up or remote monitoring (Fig. 1).

In the same-day discharge pathway, the Healthdot system continuously recorded heart rate and respiratory rate. The threshold value for an alarm was 110 bpm for heart rate (2 points) and 20/min for respiratory rate (1 point) as in accordance with the literature [1, 2]. If the warning score was 2 or higher, the eNurse contacted the patient by telephone during office hours.

The correlation between the occurrence of an alarm and postoperative complications was examined. For patients with complications, the full 7-day dataset of heart rate and respiratory rate was manually reviewed to assess whether abnormalities were visible before, during or after the complication.

## Results

A total of 679 patients were included, of whom 556 (81.9%) were female. The median age was 41 years (31–51) and mean preoperative BMI was 40.6 *±* 4.2 kg/m^2^. Roux-en-Y Gastric Bypass was performed in 628 patients (92.5%) and Sleeve Gastrectomy in 51 patients (7.5%). For context, non-eligible patients had a mean BMI of 43.9 *±* 7.4 kg/m^2^. Obesity-related complications were more prevalent among non-eligible patients (63.5%) than among eligible patients (39.9%), reflecting the selection of a relatively low-risk population for SDD. In comparison, large worldwide registry data report shows a median BMI of 41.7 kg/m^2^ and high rates of obesity-related complications, including type 2 diabetes (19.5%), hypertension (30.2%), and OSAS (18.4%) [3].

Of the 679 same-day discharge patients, 22 (3.2%) developed postoperative complications within the first week (Table 1). Four (0.6%) of these were classified as Clavien-Dindo (CD) grade *≥* 3B while the remaining 18 (2.6%) were classified as minor (CD *≤*3 A). The majority (*n* = 10) had rectal bleeding which were all treated with tranexamic acid. The four major complications included anastomotic leakage, leakage after sleeve gastrectomy suspected of micro perforation, severe postoperative bleeding and adhesiolysis due to postoperative pain. In case of the suspected micro perforation the Healthdot system generated a score of 1 on postoperative day 1 due to respiratory rate. An alarm was triggered the following morning due to increased heart rate and the patient was contacted but reported no symptoms at that time. The patient later presented with malaise complaints. For the other three major complications, no alarm was triggered prior to clinical presentation.

**Table 1:** Complications in the first seven postoperative days

Complications according to Clavien Dindo 1 2 3a 3b	2 (0.3) 15 (2.2) 1 (0.1) 4 (0.6)
Types of complications *Endoluminal bleeding* *Omental hematoma* *Omental infarction* *General malaise* *Wound problems* *Urinary tract infection* *Pulmonary embolism* (Negative) diagnostic laparoscopy* *Leakage (micro perforation)* *Intra-abdominal bleeding* *Anastomotic leakage*	11 (1.6) 1 (0.1) 1 (0.1) 1 (0.1) 1 (0.1) 1 (0.1) 1 (0.1) 2 (0.3) 1 (0.1) 1 (0.1) 1 (0.1)

Data are presented as *n* (%). Complications according to Clavien-Dindo classification

* Negative diagnostic laparoscopy: exploratory laparoscopy without identifiable intra-abdominal pathology, typically performed for unexplained postoperative pain

Analysis of the Healthdot data of these patients revealed that in several cases (*n* = 9), elevated heart rate or respiratory rate occurred prior to the complication. However, the timing and pattern of these deviations varied considerably across patients. For instance, in two cases, vital sign deviations were observed only after the complication had occurred. 7 other patients showed continuously elevated heart rates and/or respiratory rates throughout the entire 7-day monitoring period, without a clear rise during the complication. In 8 cases, no alert was triggered, either because the deviations were not simultaneous or because the threshold values were not exceeded.

**Fig. 2:**
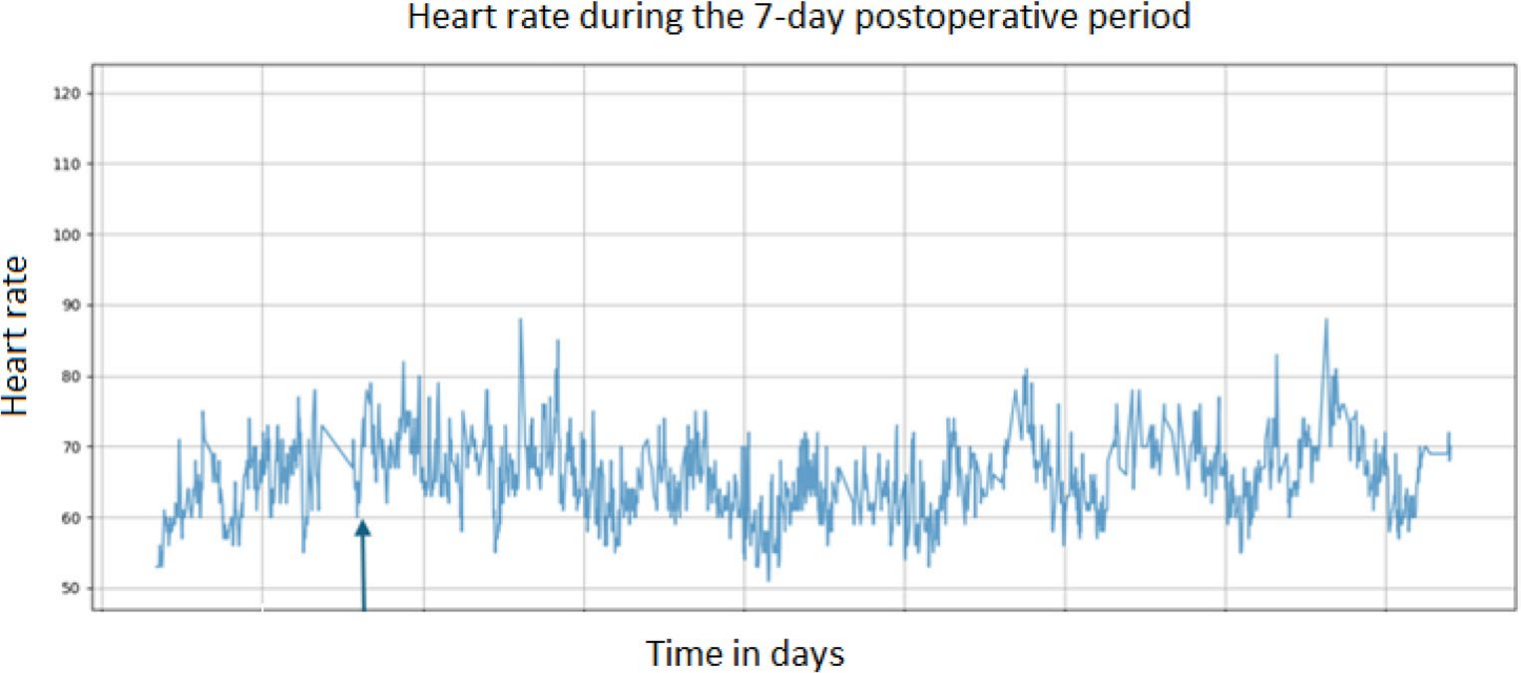
Example of heart rate trends in a patient with a complication of rectal bleeding *The arrow indicates the moment the complication occurred.*

In general, the dataset showed high variability in vital signs. Even within subgroups, such as those with rectal bleeding, no consistent pattern could be identified. Figure 2 illustrates a representative example of a patient with a complication, highlighting the lack of threshold crossing. Lowering the heart rate or respiratory rate threshold would not have consistently improved detection, as many complications showed isolated changes or deviations occurring after the complication. In addition, the low number of complications further limits the ability to define new cut-off values.

Beyond the numerical data presented above, informal feedback from both the surgical team and the patients suggest that patients are generally satisfied with the current care pathway. In early evaluations, patients reported feeling safe recovering at home. The structured follow-up by the eNurse has been mentioned as a reassuring element, offering personal contact and symptom assessment. These positive aspects of the care pathway are also described in literature in other centers [4]. While formal patient interviews are still lacking, early impressions from clinical experience and internal evaluations suggest that not only remote monitoring but especially personal contact with the eNurse enhances the overall patient experience.

## Conclusion

Although the Healthdot enables valid continuous monitoring, it does not reliably predict complications. The number of complications was very low, limiting the ability to develop predictive models based on subgroups. These findings suggest that while wearable monitoring contributes to safety, it may not be sufficient on its own. Instead, the broader perioperative care pathway appears to play a more significant role in ensuring patient safety and satisfaction. Informal evaluations and feedback from surgeons and the management team indicate that patients appreciate recovering at home in a familiar environment, feel reassured by remote monitoring and value the personal contact with the eNurse.

These findings highlight the limitations of device-centric monitoring and emphasize a shift to holistic, digitally supported care. Future research should evaluate the full care pathway, potentially through de-implementation studies or qualitative methods such as patient interviews. Key areas for optimization include proactive eNurse engagement, multimodal data use for personalized risk stratification, and continuous refinement of protocols based on real-world outcomes. This integrated approach may enhance safety, reduce admissions and improve the overall patient experience.

## Data Availability

No datasets were generated or analysed during the current study.
